# Potential benefit of (-)-epigallocatechin-3-gallate for macrovascular complications in diabetes

**DOI:** 10.1590/1414-431X20176511

**Published:** 2017-08-17

**Authors:** L. Tang, L. Li, J. Yang, C. Zeng

**Affiliations:** 1 Department of Cardiology, Daping Hospital, Third Military Medical University, Chongqing, China; 2 Chongqing Institute of Cardiology, Daping Hospital, Third Military Medical University, Chongqing, China; 3 Chongqing Cardiovascular Clinical Research Center, Daping Hospital, Third Military Medical University, Chongqing, China

**Keywords:** (-)-Epigallocatechin-3-gallate, Diabetes, Macrovascular complications, Diabetes complications, Hypothesis

## Abstract

Vascular problems are the most common complications in diabetes. Substantial evidence from epidemiological and pathophysiological studies show that hyperglycemia is a major risk factor for macrovascular complications in patients with diabetes. (-)-Epigallocatechin-3-gallate (EGCG), the major catechin derived from green tea, is known to exert a variety of cardiovascular beneficial effects. The protective effects of EGCG in diabetes are also evident. However, whether EGCG is beneficial against macrovascular complications that occur in diabetes remains unknown. Our previous studies demonstrated that treatment of EGCG inhibits high glucose-induced vascular smooth muscle cell proliferation and suppresses high glucose-mediated vascular inflammation in human umbilical vein endothelial cells. Therefore, we hypothesize that EGCG might be an effective potential candidate to reduce the macrovascular complications in diabetes.

## Introduction

The prevalence of diabetes has increased dramatically worldwide. Data from the Centers for Disease Control and Prevention show that nearly 26 million people in the United States have diabetes mellitus, with the vast majority (90–95%) having type 2 diabetes mellitus (1). Diabetes is associated with increased incidence of macrovascular disease, particularly cardiovascular disease (CVD), including coronary heart disease, stroke and peripheral vascular disease, which is the major cause of morbidity and mortality in people with diabetes (2,3). Substantial evidence has indicated that hyperglycemia is an important independent risk factor in the development and progression of diabetic macrovascular disease in patients with diabetes. Epidemiological studies also suggest that in people with type 2 diabetes, cardiovascular mortality is related to the degree of hyperglycemia (4), which was confirmed by another meta-analysis (5).

Both *in vivo* and *in vitro* studies have confirmed the contribution of high glucose to vascular injury. In cultured vascular smooth muscle cells (VSMCs), high glucose induced proliferation and oxidative injury (6,7) and inhibited apoptosis (8). In cultured endothelial cells, high glucose induced apoptosis, up-regulated vascular cell adhesion and molecule-mediated adhesiveness, and increased permeability (9–11). In animal models, high-glucose induced neointimal formation in a carotid arterial balloon injury model (7), impaired endothelium-dependent vasorelaxation (12), and enhanced the production of vascular cell adhesion molecules (13). All the above studies show that hyperglycemia is an important risk factor for diabetic macrovascular disease. Therefore, inhibition of high glucose-induced abnormal vascular injury is an important issue.

## Beneficial effects of (-)-epigallocatechin-3-gallate on cardiovascular diseases

In recent years, phytochemicals have offered promising new options for the development of more effective therapeutic strategies for cardiovascular diseases and diabetes. Teas produced from the leaves of the plant *Camellia sinensis* are widely consumed beverages throughout the world. Among the consumed teas, green tea is the best studied for its health benefits. Epidemiological studies have suggested that consumption of green tea might prevent the incidence of various cardiovascular diseases, including atherosclerosis (14). Green tea contains many biologically active polyphenolic flavonoids, commonly known as tea polyphenols (15). The major components of tea polyphenols are the catechins, a family that includes (-)-epicatechin, (-)-epigallocatechin, (-)-epicatechin-3-gallate, and (-)-epigallocatechin-3-gallate (EGCG).

As the principal constituent, EGCG is a major polyphenolic constituent present in green tea and has been known to exert a variety of cardiovascular beneficial effects (16,17). *In vitro* studies show that treatment with EGCG inhibited invasive activity of cultured human VSMC, restrained rat VSMC adhesion and migration, and repressed the angiotensin II-stimulated VSMC proliferation and adhesion molecule expression in human umbilical vein endothelial cells (18–21). The results in cultured cells were confirmed in animal studies. In the apolipoprotein E-null mice, EGCG treatment resulted in increased antioxidant capacity in local vascular tissue and systemic circulation, and reduced cuff-induced atherosclerotic plaque size (22). In spontaneously hypertensive rats, EGCG improved endothelial function, reduced systolic blood pressure, and attenuated myocardial ischemia-reperfusion injury (23).

## Beneficial effects of EGCG on diabetes

In recent years, several studies have investigated the potent anti-diabetic properties of EGCG. In diabetic animal models, EGCG delayed the onset of type 1 diabetes in spontaneous non-obese mice, reduced the increase of blood glucose levels and ameliorated the decrease of islet mass induced by low-doses of streptozotocin (24,25), and ameliorated vascular reactivity in rats and enhanced glucose tolerance in rodents (26,27). *In vitro* studies showed that treatment with EGCG improved the survival rate of isolated islets and reduced the loss of functional islet mass (28), and attenuated high glucose-induced harmful effects such as the down-regulation of the cardiac gap junction, insulin signaling blockade, embryonic vasculopathy and the expression of pro-inflammatory cytokines (29–32). Furthermore, EGCG is also beneficial against complications of diabetes. Yamabe et al. (33) found in a diabetic nephropathy rat model that EGCG ameliorated glucose toxicity and renal injury, thus alleviating renal damage caused by abnormal glucose metabolism-associated oxidative stress involved in renal lesions of diabetic nephropathy (Table 1).

**Table 1.** Biological beneficial actions of (-)-Epigallocatechin-3-gallate on diabetes and cardiovascular diseases.

Diabetes	Cardiovascular diseases
Survival rate of isolated islets ↑	Antioxidant capacity ↑
Glucose tolerance ↑	Endothelial function ↑
Vascular reactivity ↑	VSMC invasive activity ↓
Blood glucose levels ↓	VSMC adhesion and migration ↓
Insulin signaling blockade ↓	VSMC proliferation ↓
Embryonic vasculopathy ↓	Adhesion molecule expression ↓
Pro-inflammatory cytokines ↓	Atherosclerotic plaque size ↓
Lesions of diabetic nephropathy ↓	Systolic blood pressure ↓

VSMC: vascular smooth muscle cell;

↑: increase or improve;

↓: decrease or attenuate.

## Potential benefit of EGCG on macrovascular complications

A follow-up study showed the safety of EGCG in healthy individuals taking green tea polyphenol products in amounts equivalent to the EGCG content in 8–16 cups of green tea (800 mg EGCG) once a day or in divided doses twice a day for 4 weeks (34). There was a >60% increase in the systemic availability of free EGCG. This result was in accordance with other studies, which reported that the long history of green tea consumption seems to have no obvious adverse effects (35,36). Therefore, it might be possible that the extended use of green tea or EGCG by humans could build sufficient EGCG concentration in the plasma.

We propose the hypothesis that oral administration of EGCG might be a safe and effective therapeutic option to prevent/cure macrovascular complications in patients with diabetes. To test this hypothesis, we propose a systematic use of EGCG or green tea polyphenol mixture in patients with diabetes. Of course, the safety of EGCG should be verified in a diabetic animal model first. This should include genotoxic, acute and short-term toxicity, teratogenicity and reproductive toxicity (37–39), before attempting a large scale study involving human subjects.

In preliminary results of our investigations, we found that treatment with EGCG inhibited high glucose-induced VSMC proliferation by inhibiting PKC and ERK1/2 signaling in rat VSMCs (40) and suppressed high glucose-induced vascular inflammation in human umbilical vein endothelial cells by inhibition of PKC and NF-κB signaling pathway (J. Yang, Y. Han, C. Chen, D. He, L. Zhou and C. Zeng, unpublished data). This strongly supports our hypothesis regarding the beneficial effect of EGCG in macrovascular complications in diabetes.

## Cellular and molecular mechanisms of protective effect of EGCG on macrovascular complications

The underlying mechanisms of EGCG-mediated protective effects on macrovascular complications are not very clear. There are reports showing that EGCG exerts positive actions by attenuation of the apoptotic effect of cytokines, including interleukin 1β and interferon-γ, to human islet cells through the inhibition of nuclear factor-κB activation, which reduces the production of inducible nitric oxide synthase (24,25). Thus, EGCG has a protective effect on islet cells, which may attenuate high glucose-induced harmful macrovascular effects in diabetes.

Besides the effect on islet cells, EGCG might directly act to prevent/cure macrovascular complications in patients with diabetes. It has been shown that EGCG could prevent vascular reactivity through nitric oxide- and prostaglandin-dependent pathways and via attenuation of aortic lipid peroxidation in diabetic rats (26). It has also been reported that EGCG can inhibit the angiotensin II-stimulated VSMC proliferation via suppression of mitogen-activated protein kinase and nuclear transcription factor activator protein-1 signaling pathways (20). Moreover, EGCG is a potent inhibitor of VSMC adhesion by affecting integrin β1 expression and binding to extracellular matrix, and is able to inhibit laminin-induced VSMC migration (19). As VSMC proliferation, adhesion and migration are critical events in macrovascular complications, these cellular and molecular mechanisms of EGCG may be effective in the prevention of macrovascular complications in diabetes, which needs to be confirmed in the future.

## Conclusion

Based on previous findings and our preliminary data, we believe that EGCG might be a potential candidate to reduce the occurrence of macrovascular complications in diabetic patients. Confirmation of our hypothesis might provide a novel therapeutic strategy for these patients. Well-designed clinical and laboratory studies could confirm our hypothesis.
